# Cognitive behavioural therapies with a trauma focus for children and adolescents: a commentary on a systematic review and meta-analysis

**DOI:** 10.12968/jfch.2025.2.2.66

**Published:** 2025-02-02

**Authors:** A Lambert, A Doherty, J Harrison

**Affiliations:** Lancashire and South Cumbria NHS Foundation Trust; University of Lancashire

## Introduction

Evidence suggests that overall, approximately one in six children and young people (16%) will develop post-traumatic stress disorder (PTSD) after exposure to trauma (Alisic et al., 2014). Interpersonal trauma (e.g. assault) is thought to result in higher rates of PTSD than non-interpersonal trauma (e.g. accidents) and evidence indicates that girls are more likely to develop PTSD than boys (Alisic et al., 2014; Nooner et al., 2012; Dyregrov 2006). The symptoms of PTSD (at all stages of life) include intrusive thoughts, avoidance of stimuli associated with the trauma, characteristic changes in mood and cognition, and alterations in arousal (such as hypervigilance, exaggerated startle response, or sleep disturbance), causing significant impairment of social, physical, occupational and educational functioning (American Psychiatric Association (APA), 2013). In the first six months post-trauma, there is a natural recovery from PTSD in 50% of children and young people with little change in the prevalence of PTSD or symptom severity beyond six months, and recovery is unlikely without intervention (Hiller et al., 2016). Young people who have been exposed to trauma also have double the risk of developing a mental health condition, and an increased risk of future self-harm and suicide (Lewis et al., 2019). Evidence shows that cognitive behavioural therapies with a trauma-focus (CBTs-TF) are effective in improving symptoms of PTSD in children, young people and adults, and are recommended in clinical guidelines (NICE 2018; WHO 2013). However, individual clinical trials of CBTs-TF have inadequate statistical power to comment on moderators of efficacy including individual-level factors such as age, gender and type of trauma (de Haan et al., 2024). A systematic review and meta-analysis by de Haan et al. (2024) aimed to determine the efficacy of CBTs-TF for children and young people, relative to passive and active control conditions, and to examine both individual-level and treatment-level factors (such as study design and setting) that may moderate treatment effects.

## Aim of commentary

This commentary aims to critically appraise the methods used within the meta-analysis by de Haan et al., (2024) and to reflect on the relevance of the findings for clinical practice and further research.

### Results of de Haan et al. (2024)

The review conducted by de Haan (2024) identified 38 studies, of which 25 studies provided individual participant data that were included in the review’s meta-analysis. Results of de Haan et al., 20204 are summarised in Table 1.

## Commentary

A critical appraisal of the 2024 review by de Haan was assessed using the AMSTAR 2 tool (Shea et al, 2017). The review met nine out of the sixteen items in the AMSTAR tool’s criteria for assessment. The quality assessment highlighted that although a comprehensive and systematic literature search was undertaken - including unpublished studies and grey literature - there was no justification provided as to why only studies published in English were included. The review authors did not provide a list of excluded studies. Two reviewers independently evaluated risk of bias; however, it is unclear whether at least two reviewers independently performed data extraction. Furthermore, the sources of funding for the individual studies included in the review was not reported.

An individual participant data meta-analysis was performed by de Haan et al., (2024). Cochrane risk of bias ratings for the 25 studies that provided individual participant data were calculated by the review’s authors using the Cochrane Risk of Bias 2 tool (Higgins et al. 2024). Twenty of these 25 studies were scored as ‘Some Concerns’ overall. The protocol states that due to a lack of marked heterogeneity in the quality of studies, a sensitivity analysis was not undertaken. The likely impact of the risk of bias in individual studies on the overall results of the review was also not discussed by the authors; and although the review authors discussed large between-study heterogeneity in the results of the review, there was no discussion of the impact of this on the results of the review.

Overall, the systematic review by de Hann et al., (2024) provides a comprehensive summary of the available data to determine the efficacy of CBT-TF in children and young people and to investigate the moderators of efficacy at an individual and treatment level. However, some caution should be applied when interpreting the results due to the limitations discussed.

Based on the findings of de Haan et al. (2024), CBT-TF is an effective treatment for children and young people exposed to trauma with post-traumatic stress symptoms, at least up to one year. This is consistent with NICE guidelines that recommend individual trauma-focused CBT intervention or active monitoring as the first-line treatment within one month of a traumatic event for children and young people aged under 18 years, with a diagnosis of acute stress disorder or clinically important symptoms of PTSD (NICE, 2018 section 1.6.6). The findings from de Haan et al. (2024) also support previous research which demonstrates the effectiveness of CBTs-TF in adults with PTSD (Mavranezouli et al, 2020; Lewis et al, 2020; Bisson & Olff, 2021), and children and adolescents with PTSD (John-Baptiste Bastien et al. 2020; Leenarts et al. 2013; Mavranezouli et al. 2020a; Thielemann et al. 2022; Xiang et al. 2021; Xie 2024). The review also demonstrates that CBT-TF is effective for improving symptoms of anxiety and depression in children and adolescents, and those with high levels of distress may particularly benefit. Previous reviews have identified similar findings for reducing anxiety and depression (Thielemann et al. 2022; Xiang et al. 2021), or just depression (Xie 2024). In practice, the review identified that CBT-TF may be considered regardless of patient characteristics such as age, gender, trauma history and even when the patient’s caregiver cannot be involved.

These are promising findings, yet there is more to consider for application into clinical practice, particularly the views of participants and their caregivers. A systematic review of young people’s and caregivers’ experiences of CBT-TF identified that young people may be apprehensive about starting therapy and have unclear expectations about the process and therapist (Neelakantan et al., (2019). Positive perceptions however increased once treatment was underway and it was suggested that engagement challenges could be effectively addressed through sensitive pacing, extended psychoeducation and information provision, creative techniques to build a trauma narrative, and a consistent therapeutic environment (Neelakantan et al., (2019). From an organisational perspective, factors to facilitate implementation of psychosocial interventions for children with trauma exposure include strong leadership and a clear vision for trauma-informed care, comprehensive training for therapists, collaboration with patients and caregivers to anticipate potential challenges, and adaptations to local or individual needs (Powell et al. 2020). To improve the implementation of evidence-based research into child and adolescent mental health services, efforts should also be made to protect funding for evidence-based practice, engage and upskill staff on integrating evidence-based practice into care, simple processes, strong leadership and engaging with stakeholders (Peters-Corbett et al.2024).

Despite evidence that CBT-TR is effective in the management of PTSD, historically there have been lingering concerns that trauma-focused interventions may worsen patient symptoms, or result in dropout from therapy, potentially raising clinical concerns about provision (Larsen 2016). However, it was identified that a minority of participants experienced exacerbation of symptoms in a study of three separate trauma-focussed interventions, and although slower in their recovery, they still completed treatment and experienced clinically significant improvement (Larsen 2016). In addition, a meta-analysis of 40 RCTs demonstrated that dropout from trauma-focused interventions was no different to non-trauma focused interventions, with the overall level of retention suggesting such interventions are well tolerated by children and young people (Simmons et al, 2021). Simmons et al. (2021) also suggested that psychological interventions delivered in a group format were associated with less dropout than individual therapy, for children and young people with PTSD. Improvements in post-traumatic stress symptoms may also be greater for children and young people in group settings following CBT intervention (Davis et al. 2023) and CBT-TF (Thielemann et al, 2022; Xie et al., 2024). This is keeping with the NICE guidance which recommends consideration of group trauma-focused CBT intervention for the prevention of PTSD in children and young people aged 7 to 17 years if there has been an event within the last month leading to large-scale shared trauma (NICE, 2018 section 1.6.7).

Many children and young people experience delays for mental health support with 28% of those referred still on waiting lists a year later, with variations across different regions (Children’s Commissioner, 2024). Waiting list times may negatively impact on engagement with services, deter the children and young people’s families from seeking help, and negatively impact on engagement with therapy (Punton et al., 2022; Reardon et al., 2018; Sherman et al., 2009; Westin et al., 2014). The treatments they do eventually access may prove challenging for some individuals and their families, suggesting actions are needed to improve the accessibility of mental health services and the inequalities in access to services that may be experienced by some population groups such as children and young people from ethnic minorities (Lowther-Payne et al., 2023).

## Further research recommendations

Most studies included in the review by de Haan (2024) were low quality (high-risk of bias) and restricted to the English-language. Further national and international high-quality (low-risk of bias) RCTs are required to assess the clinical and cost effectiveness, accessibility and acceptability of CBT-TF interventions for children and young people with PTSD. Future trials should compare differences by population groups, trauma history and type (for example inter-personal and non-personal trauma), duration of treatment, therapist profession and include longer-term outcomes (>12 months).

The review by de Haan et al. (2024) suggests that there is currently limited evidence to demonstrate whether CBT-TF is superior to other trauma-focused psychological interventions such as Eye Movement Desensitisation and Reprocessing (EMDR) for post-traumatic stress symptoms. Therefore clinically, it is not clear which therapy should be offered first line. This is a further avenue to explore for future research. A trauma-informed approach and / or trauma-informed care also covers a broad range of definitions and interventions. Future research may therefore consider using standard reporting frameworks such as the TIDieR template for intervention description and replication (Hoffman et al., 2014).

Finally, NICE research recommendations suggest that stepped care approaches for post-traumatic stress disorder are an area for further research (NICE, 2018). Stepped care is a system of delivering and monitoring treatments where the most effective yet least resource-intensive treatment is delivered first, and patients only `step-up’ to more intensive services if clinically required *(`having the right service in the right place, at the right time, delivered by the right person’*) (Wellbeinginfo, 2024). Stepped care has been used nationally since 2008 in the Improving Access to Psychological Therapies (IAPT) programme (Wakefield et al., 2021) enabling access to evidence based psychological therapies and a reduction in depression and anxiety pre-post treatment. A recent review also highlighted evidence that stepped care may improve access to PTSD treatment and was more cost-effective than controls (Roberts and Nixon, 2023).

## Figures and Tables

**Table 1:** Results of de Haan et al., (2024)

*Study characteristics*	
Population	Cumulative total of 1686 participants aged 6-18 years, with a mean age of 13.7 years.
	63% of participants were female. Ethnicity was not described.
	Population samples varied and included: school, clinical, refugee camp, former child soldier, offender, non-governmental organisation, emergency department or mixed.
	62% of cases involved interpersonal trauma. The remainder involved accidental trauma (11%), or the case was not reported (27%).
Studies	Most studies (n=15) were from high-income countries. The remaining studies were either upper or lower-middle-income (n=7) or low-income countries (n=3).
	The included studies were assessed for risk of bias: five studies were identified as low-risk and twenty were assessed as having some concerns mainly due to un-blinding of outcome assessment (e.g. self-report or unmasked assessors) or a lack of a pre-registered protocol.
Interventions	Different types of trauma-focused CBT interventions (CBT-TF) were included: - cognitive behavioural therapy (CBT) - CBT with a trauma focus (TF) - abuse focused CBT - cognitive therapy - cognitive behavior writing therapy - developmentally adapted cognitive processing therapy - variations of narrative exposure therapy - prolonged exposure therapy for adolescents - trauma affect regulation All therapies were delivered face-to-face. The intended number of therapy sessions varied from 4 to 30. Passive control conditions were no intervention or usual care, and active control conditions were non-trauma focused psychosocial interventions such as meditation, or trauma-focused, non-CBT psychosocial interventions such as Eye Movement Desensitisation).
***Outcomes***	***Findings***
Post-traumatic stress symptoms	When adjusted for post-traumatic stress symptoms before treatment, meta-analysis of the 25 studies identified that there were significantly less post-traumatic stress symptoms after treatment with CBTs-TF compared to passive control conditions (no intervention or usual care). There was a smaller difference in effect when compared with non-trauma focused psychosocial interventions and no difference with trauma focused non-CBT psychosocial interventions (active controls). The reduction of symptoms after CBTs-TF was evident in follow-up assessments at three, six and 12 months with insufficient data to comment beyond this time-point. The efficacy of CBTs-TF interventions was increased in participants with greater severity of post-traumatic stress symptoms pre-treatment.
PTSD diagnosis	Insufficient data in the included studies to comment on PTSD diagnosis
Co-morbid disorders	CBTs-TF were associated with reductions in depression and anxiety when compared with passive control conditions (no intervention or usual care) and non-trauma focused interventions up to 12-month follow-up. There was no difference identified with trauma focused non-CBT psychosocial interventions. Higher levels of depression and anxiety before treatment improved the effect of the intervention on depression and anxiety outcomes.
	CBTs-TF reduced externalising problems compared with treatment as usual, and no treatment. However, there was no difference in externalising problems when compared to active controls. and non-CBT interventions that were trauma focused were more effective at reducing externalising problems after treatment.
Moderators	There was no evidence that individual-level factors including: age, gender, trauma type, trauma history and dysfunctional post-traumatic cognitions moderated post-traumatic symptoms after CBTs-TF or secondary outcomes. No ethnicity data was presented.
	The effect of treatment-level factors was also analysed. The greater the number of intended CBTs-TF sessions planned pre-treatment correlated with a reduction in the post-traumatic stress symptoms present post-treatment and improvement in secondary outcomes. However, following analysis on only those patients who received CBTs-TF, there was no evidence that intended duration influenced primary or secondary outcomes.
	Intended involvement of carers in CBTs-TF (prior to treatment) did not moderate post-traumatic stress symptoms after treatment and up to 12-month follow-up. Similar effects were reported for depression and anxiety outcomes. Intended carer involvement in CBTs-TF reduced externalising problems after treatment, compared to control conditions.
	There was no evidence that pre-treatment externalising of problems moderated externalising of problems post-CBTs-TF treatment.

## Key Points

▪ Evidence suggests that Cognitive Behavioural Therapy with a trauma focus (CBT-TF) is an effective intervention for children and young people exposed to trauma with post-traumatic stress symptoms, depression, anxiety or externalising problems.
▪ Individual level factors such as age, gender, trauma history and carer involvement do not have a moderating effect on post-traumatic stress symptoms, suggesting CBT-TF is suitable for young people irrespective of characteristic or caregiver involvement.
▪ CBT-TF may be more effective for children and young people with higher levels of post-traumatic stress symptoms, depression or anxiety.
▪ Further research is needed to ascertain comparison with other trauma focused psychological interventions and the likely duration of treatments’ effectiveness.

## CPD reflective questions

1) How does this review demonstrate that CBT-TF is an effective treatment for children and young people experiencing post-traumatic stress symptoms?
2) Is there any evidence that individual-level factors moderate the effects of CBT-TF?
3) What are the possible barriers to utilising CBT-TF in your clinical practice and how could these be overcome?
4) Will reading this paper change your clinical practice and why?
